# Role of Preoperative Breast MRI in Predicting Tumor-Infiltrating Lymphocytes in Breast Cancer: Is There an Association with Tumor Biological Subtypes?

**DOI:** 10.3390/biomedicines13061364

**Published:** 2025-06-02

**Authors:** Silvia Gigli, Emanuele David, Giacomo Bonito, Luisa Favale, Silvia di Sero, Antonio Vinci, Lucia Manganaro, Paolo Ricci

**Affiliations:** 1 Department of Diagnostic Imaging, Sandro Pertini Hospital, Via dei Monti Tiburtini 385, 00157 Rome, Italy; 2 Radiology Unit 1, Department of Medical Surgical Sciences and Advanced Technologies “GF Ingrassia”, University Hospital “Policlinico G. Rodolico”, University of Catania, 95123 Catania, Italy; emanuele.david@uniroma1.it; 3 Department of Emergency Radiology, Policlinico Umberto I Hospital, Sapienza University of Rome, Viale del Policlinico 155, 00161 Rome, Italy; giacomo.bonito@uniroma1.it (G.B.); paolo.ricci@uniroma1.it (P.R.); 4 Department of Radiological, Oncological and Pathological Sciences, Policlinico Umberto I Hospital, Sapienza University of Rome, Viale Regina Elena 324, 00161 Rome, Italy; luisafavale92@gmail.com (L.F.); silviadisero@gmail.com (S.d.S.); lucia.manganaro@uniroma1.it (L.M.); 5 Local Health Authority ASL Roma 1, 00193 Rome, Italy; antonio.vinci.at@hotmail.it

**Keywords:** breast cancer, magnetic resonance imaging, tumor-infiltrating lymphocytes, immunotherapy, apparent diffusion coefficient

## Abstract

**Introduction:** A potential prognostic biomarker for predicting the response to immunotherapy in breast cancer (BC) is tumor-infiltrating lymphocytes (TILs). The purpose of this research is to examine if preoperative characteristics of breast magnetic resonance imaging (MRI) may be used to predict TIL levels in a group of BC patients. In addition, we aimed to assess any potential relationship between the various tumor biology subgroups and MR imaging characteristics. **Materials and Methods:** This retrospective analysis comprised 145 participants with histologically confirmed BC who had preoperative DCE MRI. We collected and examined patient information as well as tumor MRI features, such as size and shape, edema, necrosis, multifocality/multicentricity, background parenchymal enhancement (BPE), and apparent diffusion coefficient (ADC) values. We divided patients into two groups based on their TIL levels: low-TIL (<10%) and high-TIL groups (≥10%). Following core needle biopsy, tumors were categorized as Luminal A, Luminal B, HER2+, and Triple Negative using immunohistochemical analysis. TIL levels were correlated with tumor biological profiles and MRI features using both parametric and non-parametric tests. **Results:** Patients were categorized as having a high TIL level (≥10%; 54/145 patients) and a low TIL level (<10%; 91/145 patients) based on the median TIL level of 10%. Of the lesions, 13 were HER2-positive, 16 were Triple Negative, 49 were Luminal A, and 67 were Luminal B. Higher TIL levels were statistically correlated with TNBC (11/16 individuals, *p*: 0.007). ADC values (*p* = 0.01), BPE levels (*p* = 0.008), and TIL levels were all significantly negatively correlated. Significantly more homogenous enhancement was seen in tumors with elevated TIL levels (*p* = 0.001). The ADC values and the enhancing characteristics were the most important factors in predicting TIL levels, according to logistic regression analysis, and when combined, they demonstrated the strongest ability to distinguish between the two groups (AUC = 0.744). **Conclusions:** MRI features, particularly ADC values and enhancement characteristics, may play a pivotal role in the assessment of TIL levels in BC before surgery. This could help patients to better customize treatments to the features of their tumors.

## 1. Introduction

The use of immunotherapy for aggressive forms of breast cancer (BC) represents a relatively recent approach compared to traditional treatments such as surgery, chemotherapy, and radiotherapy, offering long-lasting clinical responses and improved survival rates [1,2,3].

Several authors have shown that a significant percentage of Triple Negative breast cancer (TNBC) patients can benefit from the use of immune checkpoint inhibitors (ICBs), especially in cases of programmed cell death-ligand 1 (PD-L1) expression [4].

Lately, recent studies have demonstrated that antitumor immunity influences the efficacy of HER2-targeted therapy, highlighting the potential role of anti-PD1 therapy as a therapeutic target in HER2 + BC subtypes [5]. The immune response in luminal BC is lower compared to other BC groups. This could be related to different biological characteristics, including a lower number of genetic mutations and a reduced infiltration of immune system cells [6]. Ongoing research aims to assess whether specific subgroups of luminal patients may see improvements in the effectiveness of treatments by using immunotherapy; several studies are exploring the use of immunotherapy in combination with targeted therapies to enhance the immune response in such cases [7].

Unfortunately, the efficacy of immunotherapy is achieved in a limited subset of patients. In addition, these therapies could have serious adverse effects, including autoimmunity, emphasizing the critical role of predictive biomarkers in identifying suitable candidates [8].

Pre-existing anti-tumor immunity is critical to the success of ICB-based immunotherapy; a promising immunologic marker in this setting is the number of tumor-infiltrating lymphocytes (TILs) [9].

Intratumorally, TILs are lymphocytes located inside tumor nests, directly contacting carcinoma cells via cell-to-cell interaction. On the other hand, stromal TILs do not come into direct contact with cancer cells; instead, they are dispersed throughout the stroma between carcinoma cells.

The current guidelines from the International Immuno-Oncology Biomarker Working Group on Breast Cancer propose that TIL assessment should be conducted in the stromal rather than intraepithelial compartments. This recommendation’s justification arises from the finding that intratumoral TILs are generally found in smaller quantities, exhibit greater heterogeneity, and are harder to identify on H&E slides without additional techniques such as immunohistochemistry or immunofluorescence [9,10].

The degree of lymphocytic infiltration has been shown to have predictive and prognostic value in BC, and is related to the adaptive immune response, especially in TNBC and HER2-positive BC [11,12,13,14]. Numerous studies have shown that immunological characteristics, such as stromal TILs, are associated with increased rates of pathological complete response (pCR) in patients receiving neoadjuvant chemotherapy (NAC) [9,15].

The role of magnetic resonance imaging (MRI) has evolved within oncological practice in recent years. MRI-based imaging biomarkers can be used to examine a variety of biological tissue characteristics, such as tumor microstructure, metabolism, and composition.

The usefulness of MRI characteristics to estimate TIL levels in breast cancer has been examined in earlier research [16,17,18], although most studies focused only on TNBC and HER2+ cases, excluding luminal BC.

Choi, W.J. et al. investigated the relationship between MRI features and tumor-infiltrating lymphocytes (TILs) in estrogen receptor (ER)-negative, HER2-positive breast cancer and found that certain MRI characteristics, such as lesion enhancement patterns, were significantly correlated with higher levels of TILs [16]. Wu, J et al. explored associations between MRI features and found correlations between imaging features (e.g., lesion texture, morphology) and gene expression profiles related to immune activity. This study emphasized the value of integrating imaging with molecular data for the non-invasive evaluation of TILs [18]. Our study aims to investigate the potential role of preoperative breast MRI features—including a range of morphological, enhancement, and apparent diffusion coefficient (ADC) parameters—in predicting tumor-infiltrating lymphocyte (TIL) levels in patients with breast cancer. Furthermore, the study seeks to evaluate the association between these MRI characteristics and the various biological subtypes of breast cancer, including luminal subtypes.

## 2. Materials and Methods

### 2.1. Study Population

We performed a retrospective study including patients who were referred to the Department of Radiology, Oncology, Pathological Anatomy at Policlinico Umberto I hospital, Sapienza University of Rome, Italy, in the period between September 2022 and August 2024.

The study was conducted in accordance with the Declaration of Helsinki; being a retrospective study, all patient information was de-identified and patient consent was not required.

The following requirements for inclusion were met by every patient:

- Diagnostic core needle biopsy confirms the diagnosis of invasive BC;
- Preoperative breast MRI performed at our department with full MRI data;
- Availability of the final histological analysis on the surgical specimen, which includes the tumor biological profile (ER and PgR status, HER-2 status) and Ki67 status;
- No prior history of surgical, radiant, or neoadjuvant chemotherapy breast treatment prior to the MRI examination

All MRI exams were performed before the core needle biopsy. In the case of an incomplete/inadequate tumor biological profile (on the core needle biopsy histological diagnosis), patients were excluded. Patients with low image quality and those who did not have surgery in our surgical department were also ruled out.

Finally, 145 breast cancer patients (mean age 56.9 ± 12.4 years; range, 32–89 years) were eligible for the proposed study. Clinical data, including patient age, familiarity of BC, exposure to hormone therapies, and menopausal state, were collected.

### 2.2. MRI Examination and Evaluation

All MRI exams were performed on a 3T magnet (Discovery 750; GE Healthcare, Milwaukee, WI, USA) in prone positions using a dedicated eight-channel breast coil. Images were acquired following this protocol:

- T2-weighted axial single-shot fast spin echo sequence with a modified Dixon technique (IDEAL) for intravoxel fat–water separation (TR/TE 3500–5200/120–135 ms, slice thickness 3.5 mm).
- Diffusion-weighted axial single-shot echo-planar sequence (TR/TE 2700/58 ms, slice thickness 5 mm) with a diffusion-sensitizing gradient with a b-value of 0, 500, and 1000 s/mm^2^.
- Dynamic 3D-T1w axial and sagittal gradient echo sequence with fat suppression after injection of 0.1 mmol/kg body weight of Gadoteric acid (Dotarem^®^, Guerbet S.p.A, Villepinte France, or Claricyclic^®^, GE Healthcare S.r.l., Chicago, IL, USA) at a rate of 2 mL/s, followed by a bolus of 15 mL saline flush (TR/TE 4/2 ms, slice thickness 2.4 mm) before and five to ten times after intravenous contrast medium injection.

For a more detailed tumor analysis, post-processing automatically generated subtracted images from the images following the administration of the contrast medium. A region of interest (ROI) was placed within the region on a subjectively identified area of maximal contrast enhancement, and all of the obtained Digital Contrast Enhanced (DCE) series were evaluated to automatically generate a signal intensity-to-time curve (SI/T) for each index lesion. According to the most recent ACR Breast Imaging Reporting & Data System (BI-RADS) guidelines, the kinetic curves were categorized as I (progressive wash-in), II (plateau), or III (wash-out) [19,20]. Blinded to clinicopathologic data, two radiologists with 25 and 10 years of experience in breast imaging evaluated each MRI examination. DCE sequences were used as reference images for tumor detection and lesion characterization, gathering the following MRI characteristics for each lesion:

- Size (mm) and shape;
- Type of enhancement (mass/non-mass like);
- Intralesional enhancement: homogeneous, heterogeneous, or rim;
- Tumor-associated edema on T2-weighted images;
- Intratumoral necrosis;
- Tumor multifocality/multicentricity (in these situations, the statistical analysis included the largest lesion, which was regarded as the index one);
- Axillary lymph-node involvement;
- Level of background parenchymal enhancement (BPE) according to the BI-RADS lexicon.

The subtracted images were overlaid on the ADC map to assess the ADC values for quantitative research. A manually drawn ROI with a diameter of 3–6 mm was placed on the slide where the lesion reached its greater diameter. The ADC value was automatically generated and stored. To avoid regions of T2 shine-through, such as the necrotic cores, ADC measurements were limited to the enhanced solid portion of the tumor.

### 2.3. Histologic Evaluation

Following staging, all patients had either a mastectomy or breast-conserving surgery, and the surgical specimen’s largest diameter of the identifiable tumor mass was measured. The WHO classification was used for the histopathological diagnosis, and the Nottingham Histological Grading Score (NGS) was used to assess the tumor grade. Nuclear pleomorphism, mitosis, and the degree of tubular formation are the three parameters that the NGS rates on a scale of 1 to 3. The sum of the three parameters’ individual scores determines the final histological grade: 3, 4, or 5 indicates grade 1; 6 or 7 grade 2; and 8 or 9 grade 3 (3, 7).

The International Immuno-Oncology Biomarker Working Group on Breast Cancer guidelines [15] were followed in evaluating the levels of stromal TILs; in each instance, the proportion of lymphoid cells to stroma within the tumor was noted as a percentage.

To determine the Ki-67 index and assess the status of the molecular receptors (ER, PgR, and HER2), immunochemical (IHC) analysis was carried out. IHC analysis was used to evaluate ER and PgR using a 1:100 dilution of the Dako monoclonal antibody. The Ki-67 index was evaluated using the monoclonal antibody Mib-1 (1:200 dilution; Dako, Glosturp, Denmark). Samples with an equivocal IHC result were assessed for HER2 status using fluorescence in situ hybridization (FISH) analysis. There were five different HER2 expression scores: 0, 1+, 2+, and 3+. Only tumors that scored 3+ were considered HER2-positive.

Tumors were classified into four molecular subtypes using estrogen and/or progesterone receptors and HER2 status, as determined by immunohistochemistry (IHC) or fluorescence in situ hybridization (FISH) and Ki67. HER2 was classified as positive by either an IHC score of three and/or FISH amplification. The biological subtypes included the following:

- Luminal A;
- Luminal B;
- Triple Negative (TN);
- HER2+.

Human epidermal growth factor receptor 2 expression and a lack of hormonal receptors are characteristics of the HER2+ subtype, whereas the luminal subtypes are distinguished by the expression of hormonal (estrogen and/or progesterone) receptors. Lastly, the lack of HER2 and hormonal receptors is a characteristic of the TN subtype. Additional classifications for luminal subtypes include Luminal A (HER2− and Ki67 < 14%) and Luminal B (HER2+/−, Ki67 > 14%) based on the expression of HER2 and the proliferation rate of Ki67 [20].

### 2.4. Statistical Analysis

While qualitative variables were expressed as absolute numbers and percentages, quantitative variables were represented as means and standard deviations. The statistical software Stata^®^ v.17.0 (Stata Corp., Collegue Station, TX, USA) was used to perform the statistical analysis, while MS Excel vs. 2504, MS Visio vs. 365, and Stata v.17.0 were used for graph drawing. G*Power software v. 3.1.9.7 was used for post hoc power analysis and visualization. The statistical significance threshold was set at *p* < 0.05 for most inferential analyses, with the following exceptions:

- *p* = 0.10 was used for candidate predictor variables in the regression model;
- *p* = 0.025 (Bonferroni correction) was used for the OR of the final proposed model, which uses two predictor radiological variables;
- *p* = 0.00625 (Bonferroni correction) was used for assessing the reliability of the proposed model as a whole, accounting for all eight radiological variables that were screened for the model after the exploratory analysis.

For continuous variables, we used an independent t test or a Mann–Whitney U test to compare the two groups. The Homogeneity of Variances test (Levene’s test) and the Normality test (Shapiro–Wilk test) were performed to verify the hypotheses.

The relationships between high/low-TIL groups and MRI parameters were investigated using a series of linear regressions. Candidate predictor variables (*p* < 0.10) were used to construct a multivariate logistic model, while other variables were discarded as non-explicative.

For the logistic model, since it necessitates a dichotomous (yes/no) variable, TIL presence was classified as either high TIL or low TIL, with a cut-off of 10%. This cut-off was proposed based on the median TILs of the sample. Once the final model was constructed, Receiver Operating Characteristic (ROC) curve analysis was employed for both univariate predictive variables and the whole multivariate model, assessing the Area Under the Curve (AUC) and graphing the results. Youden’s index was used to determine the optimal thresholds, and then the sensibility, specificity, and accuracy of the final model was determined. Internal validation was assessed with bootstrapping (1000 replications); bootstrap p-values and confidence intervals were reported. A post hoc power evaluation was conducted using G*Power software, using the observed values to determine the power level reached in the analysis. Finally, a decision flowchart was proposed, with interpretation for expected TIL levels given the radiological findings.

## 3. Results

### 3.1. Clinicopathologic Features

A total of 145 patients were eligible for the study (mean age 57 years, range 32–78 years); their characteristics are summarized in Table 1. A total of 58 were of childbearing age, 87 were in menopause, 20 had previously taken hormonal therapies, and 51 had a family history of breast cancer.

Of 145 breast cancers, 97 (66.8%) were no special type, 42 (29.0%) were lobular, 2 (1.4%) were tubular, 2 (1.4%) were mucinous, 1 (0.7%) was apocrine, and 1 (0.7%) was neuroendocrine.

Regarding tumor grade, 25 were of a low grade (G1), 82 (56.6%) were of an intermediate grade (G2), and 38 (26.2%) were of a high grade (G3).

Despite the recommendation to assess TILs on a continuous scale, there is disagreement over a threshold for TILs that is clinically significant [15]. Using the median TIL value in the study population as a cut-off (median TILs level 10%) we divided patients into two groups to compare the data and ascertain whether there were any notable differences between tumors with high and low levels of TILs as follows:

- Patients with high TIL levels (≥10%; 54/145 patients).
- Patients with low TIL levels (<10%; 91/145 patients).

Histological subtypes were distributed as follows: A total of 49 lesions were classified as Luminal A, 67 as Luminal B, 13 as HER2-positive, and 16 as Triple Negative.

The TNBC Group’s average tumor stage at diagnosis was stage IIIA, while the n-TNBC Group’s was stage IIB. We observed a statistical correlation between higher TIL levels and TNBC (11/16 patients, *p*: 0.007).

**Figure 1:**
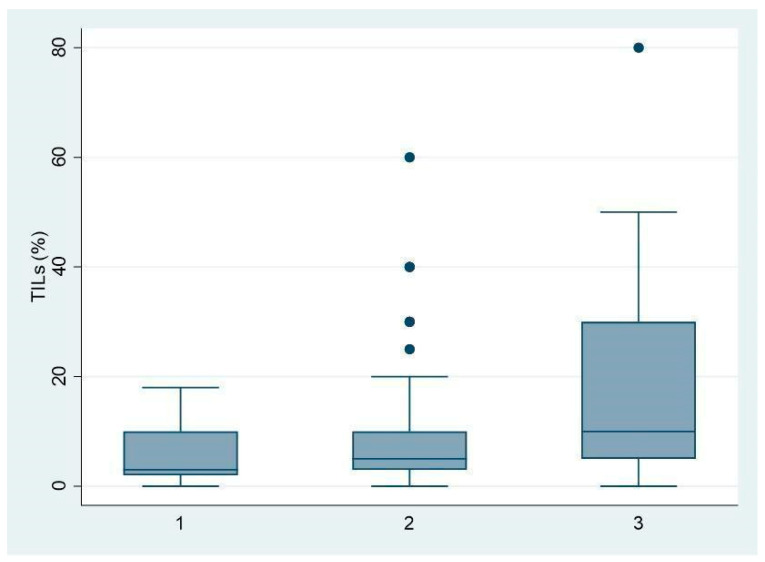
Associations between TIL levels (expressed as %) and histologic grade of BC (G1-2-3).

In addition, we found a direct statistical relationship between high-grade tumors and higher TIL levels compared to low-grade tumors (*p* = 0.005; grade 3 in 22/54 vs. 16/91), as shown in Figure 1.

Particularly, we stratified every biological subclass according to tumor grade and we found that in Luminal B BC, 20/67 cases were G3 and 18 of them had high TILs, and these data were statistically significant (*p* = 0.002).

Regarding histological tumor type, size, axillary lymph node metastasis, and multifocality, no significant statistical differences were found between the two groups (all *p* > 0.05).

The age of patients, family background of breast cancer, and menopausal status were similar in both groups (all *p* > 0.05).

### 3.2. MRI Features

We found statistically significant differences between the two groups (high/low-TIL groups) and some MRI features.

**Figure 2:**
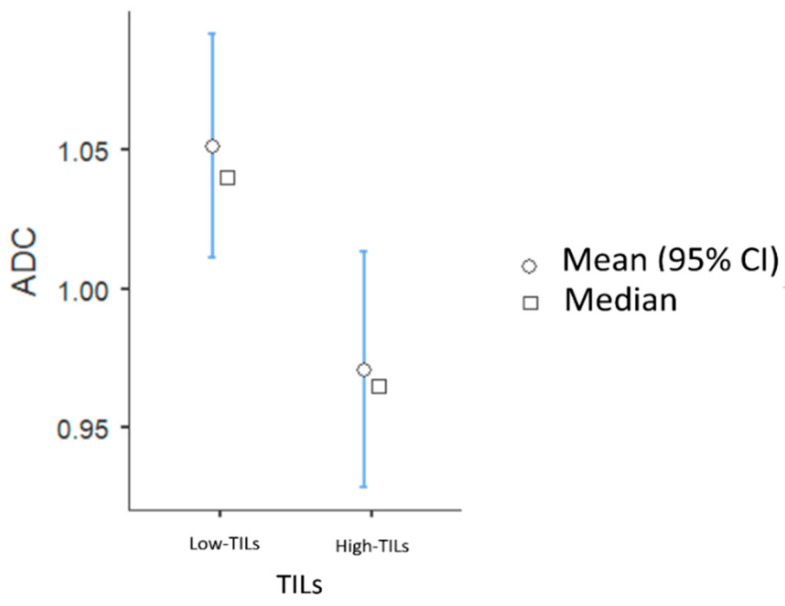
Associations between TIL levels and ADC values.

The high-TIL group showed lower levels of BPE compared to the low-TIL group (BPE 1 in 34/54 patients [62.3%] vs. 30/91 patients [33.7%], *p* = 0.008) and significantly lower ADC values (mean 0.97 vs. 1.05 × 10^−3^ mm^2^/s, *p* = 0.01) (Figure 2).

**Figure 3:**
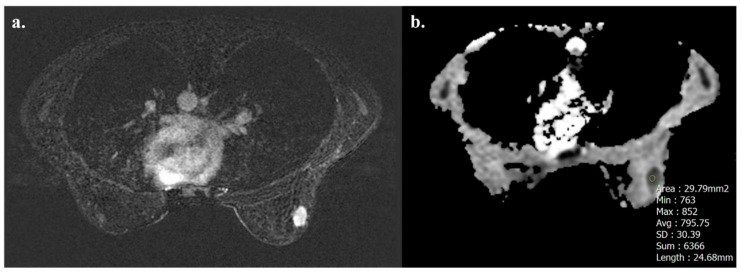
A 32-year-old woman with NST BC subtype Luminal B with high TIL levels (35%) in the right breast. Axial T1-weighted contrast-enhanced MR image shows homogeneously enhanced mass (**a**), and hypointensity on ADC map (0.795 × 10^−3^ mm^2^ s^−1^), as showed by the circle (**b**).

**Figure 4:**
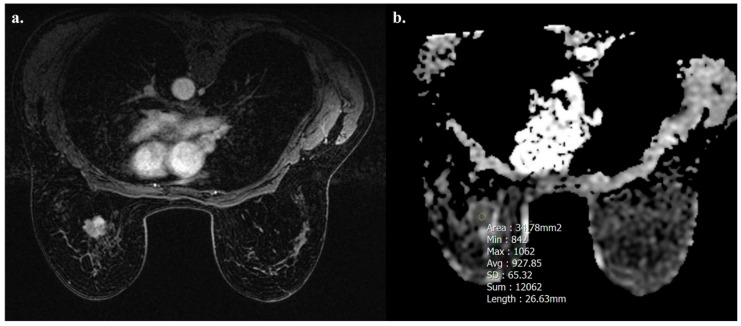
A 52-year-old woman with NST BC subtype Luminal B with low TIL levels (3%) in the left breast. Axial T1-weighted contrast-enhanced MR image shows heterogeneously enhanced mass (**a**), and hypointensity on ADC map (0.927 × 10^−3^ mm^2^ s^−1^), as showed by the circle (**b**).

Tumors with high TIL levels also showed more frequently homogeneous enhancement compared to those with low TIL levels (31/54 [57.4%] vs. 17/91 [18.7%], *p* < 0.001) (Figure 3 and Figure 4).

We did not find any other significant associations between MRI features and TIL levels in the two groups.

**Figure 5:**
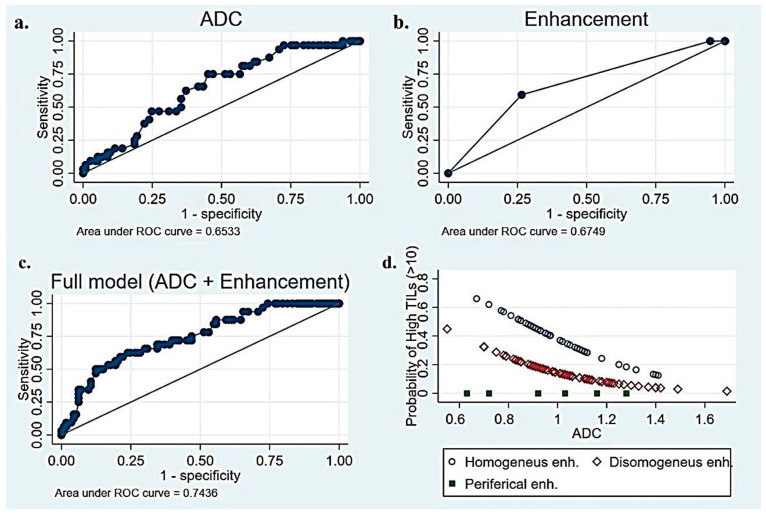
(**a**) Receiver Operating Characteristic (ROC) curve of TILs–ADC to predict the level of TILs in BC patients, (**b**) ROC curve of TILs–enhancement to predict the level of TILs, (**c**) ROC curve of combined full model (ADC + enhancement)–TILs to predict the level of TILs, and (**d**) correlation between TILs and combination of ADC–intratumoral enhancement.

**Figure 6:**
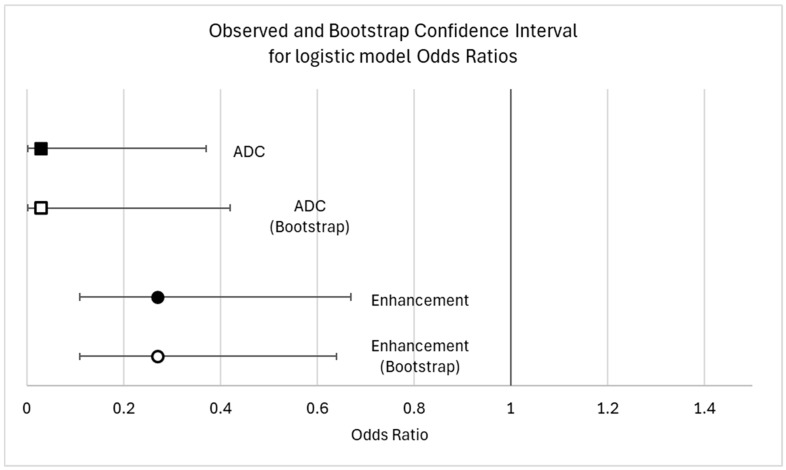
Observed and bootstrap confidence interval for model odds ratio.

The univariate linear regression results for all candidate predictor variables are available in the Supplementary Materials (Table S1). We found three independent variables associated with the percentage of TILs (*p* < 0.05). A high histologic grade (*p* = 0.002) and homogeneous intratumoral enhancement (*p* = 0.001) were positively correlated with TILs. A histologic grade of 3 was associated with an average of 11.5% more TILs (CI: 4–20), and heterogeneous enhancement correlates with 10% fewer TILs than homogeneous enhancement. The ADC values turned out to be negatively correlated with TILs (*p* = 0.019). Other variables that were positively correlated with TILs, but to a lesser degree, were successively investigated in logistic regression. The results from univariate and multivariable logistic regressions are summarized in Table 2. The ADC values and enhancement have proven to be the two most effective variables in categorizing patients into high- and low-TIL groups. As illustrated in Figure 5, the AUC for the ADC and enhancement was quite unsatisfactory (0.65 for ADC, 0.67 for intratumoral enhancement). However, the combination of the two parameters yielded a moderately higher AUC (0.74). Specifically, the predictiveness of the ADC for the TIL class (high/low) was strongly influenced by the type of observed enhancement. Rim enhancement consistently correlated with low TIL levels, whereas higher TIL levels were more likely associated with homogeneous enhancement (OR 3.7, 95% CI 1.56–8.51). The model was internally validated with the sample bootstrap method, using 1000 random resamplings (with replacements) of the original data. The bootstrap results are shown in Figure 6. Post hoc power analysis was conducted by running G*Power on the observed model parameters (adj. R^2^, alpha level, total sample size, observed OR of 3.7 for high TIL levels in homogeneous enhancement, and observed mean and standard deviations of low TIL probability). Its results suggest that 80% power was already reached with ≈ 110 included subjects.

The bootstrap estimation did show a high robustness in the proposed logistic model, with a bootstrap OR and confidence interval almost perfectly overlapping with the main analysis ones. The bootstrap odds ratio was 0.029 for the ADC (95%CI: 0.0017–0.49) and 0.27 for heterogeneous enhancement (95%CI: 0.11–0.62) (Figure 6).

**Figure 7:**
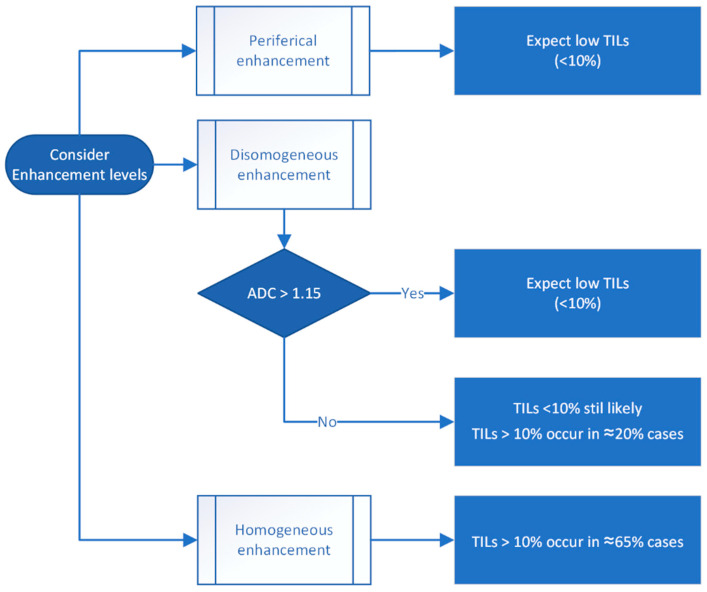
Proposed decision tree for estimating tumor-infiltrating lymphocyte (TIL) levels based on MRI enhancement patterns and apparent diffusion coefficient (ADC) values.

A step-by-step flowchart illustrating the proposed decision-making process based on MRI enhancement features and ADC values is presented in Figure 7. The decision tree integrates qualitative enhancement characteristics (and quantitative ADC measurements to stratify tumors into low- or high-TIL categories. This approach aims to provide a non-invasive tool to support the early immune profiling of breast cancer, potentially guiding treatment planning.

## 4. Discussion

Growing evidence has confirmed the clinical impact of TILs in determining prognosis in breast cancer [10,11,12,13]. We found an inverse correlation between BPE and TIL levels in our sample. This finding is consistent with previous studies. In their work, Wu et al. demonstrated higher levels of TILs in patients with low background parenchymal enhancement, while TIL levels were significantly lower in patients with high BPE. In their sample of 110 women, Choi et al. found a significant association between high TIL levels and low peak enhancement and BPE. This correlation may be related to the impact of hormonal factors both on BPE and TIL infiltration; for instance, estrogen levels can influence breast density and immune response [18].

Additionally, we found a statistical correlation between high TIL levels in our sample and homogeneous intratumoral enhancement. In their study involving 158 BC patients, Celebi et al. [21] discovered that tumors with high TIL levels exhibited more homogenous enhancement in the internal enhancement pattern (*p* = 0.001) compared to tumors with low TIL levels.

In our study, we used a 10% cut-off for stromal TILs, aligning with other studies [22,23,24], and we observed a statistically significant difference in the ADC values between high- and low-TIL groups. Our results were quite reliable on internal validation, as the model was robust to bootstrap analysis. Ku et al. [17] found no statistically significant variations in the ADC values when comparing high- and low-TIL groups, but their study included only TNBC, using a cut-off value of 50% to stratify patients.

According to some earlier research, tumors with low TIL levels had ADC levels that were noticeably lower than those with abundant TILs [23,25]. It is interesting to note that our results differed from these in that the TIL levels were inversely related to the ADC levels. However, our findings are in line with research by Lee et al. and Bian et al., which found that BC with higher TIL levels typically had lower ADC values [24,26]. These discrepancies could be linked to differences in DWI techniques, size, placement of ROIs, or study populations, as in some cases, non-mass-like lesions were excluded [23,25].

Moreover, we found that the ability of the ADC to predict the TIL class (high/low) was significantly related to the type of enhancement. The combination of these two variables showed the best ability to discriminate between the two groups (AUC 0.74) in our series.

The uncertainty surrounding the role of the ADC compared to other predictive markers highlights the need for further studies using standardized protocols to provide clarity.

In addition, we found that high histological grades were associated with increased levels of TILs, indicating that breast cancers with elevated levels of TILs tend to be more aggressive and have a reduced in situ component [24,27,28]. This correlation suggests a potential interaction between the histological characteristics of the tumor and the host immune response. Tumors with higher histological grades often exhibit aggressive biological behavior, and the immune system may respond more robustly in such cases.

Bian et al. [26] found a significant correlation between TILs and tumor size; specifically, tumors with high TIL levels generally exhibited a smaller size compared to those with low TIL levels. However, in our study, no significant difference was observed between the two groups; this may be related to the different TIL thresholds used, as Bian et al. stratified patients using a TIL cut-off of 50%.

A large percentage of the previous research excludes luminal subgroups that often do not benefit from immunotherapy in favor of focusing on HER2+ and Triple Negative cancers. The number of TILs varies according to the type of breast cancer; TNBC and HER2-positive cases show higher levels of TILs. Their function in HER2-negative (ER + /HER2-) and luminal (estrogen receptor-positive) BC is nevertheless still unknown.

In our study, we also included patients with luminal BC, but we observed a statistical correlation relationship only between higher TIL levels and TNBC (11/16 patients, *p*: 0.007). Luminal BC generally showed low TIL levels, with limited prognostic impact. This is probably related to the fact that luminal cancers typically have a less inflamed tumor microenvironment compared to other subgroups. Estrogen signaling in luminal cancers can create an immunosuppressive setting by upregulating anti-inflammatory cytokines and downregulating immune-stimulatory pathways [29,30]. However, in our sample, by stratifying every biological subclass according to tumor grade, we found that in Luminal B BC, 20/67 cases were G3 and 18 of them had high TIL levels; these data were statistically significative (*p*: 0.002). This is probably related to the complex direct relationship between tumor grade and TIL levels. High tumor grades are more likely to exhibit genomic instability, leading to stronger immune infiltration.

Recent research has investigated whether a radiomic model can accurately predict the status of TILs. Su et al. [30] further opened the way for precision therapy in breast cancer by proving that such a model is feasible in TNBC and offering a method to make its features interpretable.

The potential clinical application of our method lies in its ability to provide a non-invasive estimation of TIL levels from preoperative breast MRI, which could support the early immunological profiling of tumors. While our study does not replace histopathological evaluation, it suggests that certain MRI features—especially when combined (morphological, enhancement, and ADC parameters)—may serve as imaging biomarkers that correlate with immune activity. In the future, this could aid in stratifying patients for immunotherapy or tailoring neoadjuvant treatment strategies, particularly in subtypes like TNBC, where TIL levels have prognostic and predictive value.

There are several limitations to our investigation. First, it can be exposed to selection bias because it is a retrospective study. Consequently, it is essential to carry out additional independent external validation through successive patient recruitment and data collection. The study’s reliance on a single MRI procedure and relatively small cohort size, along with the fact that the patient cohort is drawn from a single-center institution, raises questions regarding the study’s representativeness. The reproducibility and dependability of this process are challenged by the lack of a standard for designating Regions of Interest (ROIs) for ADC data and kinetic curve analysis. In conclusion, multicenter studies with a greater number of patients should be used to further confirm the current work’s findings to improve their generalizability and robustness. To validate the results in a subtype-specific way, more research is required.

## 5. Conclusions

In conclusion, histologic grade 3, low ADC values, homogeneous enhancement, and low BPE have been identified as factors associated with high TIL levels. The combination of ADC and enhancement characteristics showed the best ability to discriminate between the two groups, resulting in an AUC of 0.74. Notably, the ability of the ADC to predict the TIL class (high/low) is significantly impacted by the type of enhancement observed.

Our study fits in with the broader landscape of precision medicine by seeking non-invasive methods for characterizing the tumor microenvironment. The integration of MRI-based assessments of TIL levels could serve as an adjunct to traditional diagnostic and prognostic approaches to guide more informed treatment decisions and contribute to the efforts to tailor therapies based on tumor characteristics, ultimately improving patient outcomes.

## Figures and Tables

**Table 1:** Patients’ characteristics.

**Age (mean, SD)**	56.8 (12.4)
**Post menopause (n, %)**	87 (60%)
**Localization (n, %)**	
UQQ	50 (34.72%)
UIQ	16 (11.11%)
QII	9 (6.25%)
LOQ	10 (6.94%)
LIQ	15 (10.42%)
LQ	9 (6.25%)
IQ	5 (3.47%)
OQ	16 (11.11%)
RT	14 (9.72%)
**Type (n, %)**	
Luminal A	53 (36.55%)
Luminal B	80 (55.17%)
HER-2	3 (2.07%)
TN	9 (6.21%)
**Grade (n, %)**	
1	25 (17.24%)
2	82 (56.55%)
3	38 (26.21%)
**TILs (mean, SD)**	9.72 (13.02)
**TILs > 10% (n, %)**	32 (22.07%)

Main features of the population, tumors detected and percentages of TILs expressed are highlighted in bold text.

**Table 2:** Results from univariate and multivariable logistic regression. In bold, statistically significant values.

UNIVARIATE LOGISTIC MODEL	OR	95% CI	*p*-Value
ADC	0.04	0.03–0.05	**0.01**
Kinetic Curve			0.22
I	1 (baseline)		-
II	1.60	0.41–6.23	0.49
III	2.75	0.71–10.51	0.14
Enhancement			**0.01**
Homogeneous	1 (baseline)		-
Heterogeneous	0.27	0.12–0.61	**0.01**
Rim	0.01	0.01	**0.02**
Edema			0.83
Absent	1 (baseline)		-
Peri-tumoral	0.7741935	0.29–2.01	0.59
Pre-pectoral	0.6857143	0.08–6.19	0.74
Subcutaneous	2.285716	0.36–14.59	0.38
More than 1 component	1.714286	0.15–19.85	0.67
Margins			0.34
Regular	1 (baseline)		-
Irregular	0.625	0.11–3.59	0.60
Lobular	0.91	0.12–6.71	0.91
Spiculated	1.10	0.18–6.57	0.92
Non-mass	0.25	0.03–2.24	0.22
Size	0.99	0.96–1.02	0.67
Stadiation			0.41
Unifocal	1 (baseline)		-
Multifocal	0.60	0.20–1.81	0.38
Multicentric	0.63	0.24–1.66	0.35
Bilateral	0.01	-	-
BPE			0.49
I	1 (baseline)		-
II	0.52	0.21–1.28	0.16
III	0.70	0.20–2.38	0.56
IV	0.44	0.05–3.87	0.46
**MULTIVARIABLE LOGISTIC MODEL**	**0.01**		
ADC	**0.01**		
Enhancement			
Homogeneous	1 (baseline)		-
Heterogeneous	0.27	0.11–0.64	**0.01**
Peripheral	0.1	-	0.99
Constant	20.77	1.43–301.13	0.02
**MODEL PARAMETERS**			
Model *p*-value	0.0001		
Sensibility	0.75		
Specificity	0.63		
Accuracy	0.75		

Statistically significant *p*-values are highlighted in bold.

## Data Availability

The raw data supporting the conclusions of this article will be made available by the authors on request.

## Supplementary Materials

The following supporting information can be downloaded at: https://www.mdpi.com/article/10.3390/biomedicines13061364/s1, Figure S1. TIL distribution by menopause condition and tumor type; Table S1. Univariate linear regression results for TILs and several candidate predictor variables, and association with histopathological findings. Statistically significant results (*p* < 0.05) are in bold.
