# Supplementary material for: Role of Preoperative Breast MRI in Predicting Tumor-Infiltrating Lymphocytes in Breast Cancer: Is There an Association with Tumor Biological Subtypes?
Source: Biomedicines. 2025 Jun 2;13(6):1364. doi: 10.3390/biomedicines13061364 (PMC12189808; doi:10.3390/biomedicines13061364)
Supplement: Supplementary file 1 [file biomedicines-13-01364-s001.zip › biomedicines-3619428-supplementary.pdf]

Supplementary Materials

Figure F1. TIL distribution by menopause condition and tumor type.

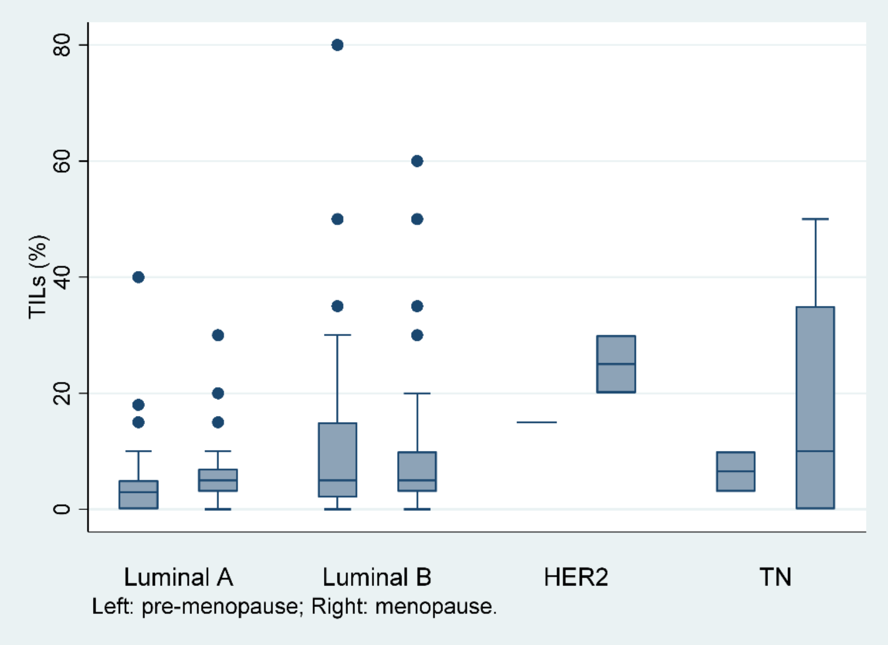

Table S1. Univariate linear regression results for TILs and several candidate predictor variables, and association with histopathological findings. Statistically significant results (p<0.05) are in bold.

Variable	p-value
Side	0.47
Localization	0.63
Age	0.35
Age (quadratic correction)	0.11
Familiarity	0.82
**Hormonal therapy**	**0.05**
Menopause	0.84
Visibility T2	0.11
Visibility DWI	0.79
**ADC**	**<0.01**
**Enhancement**	**<0.01**
Curve (rapid vs. tardive emptying)	0.03
Regular Margins	0.31
Dimensions	0.66
Stadiation (multicentric, multifocal, unicentric)	0.44
BIRADS	0.44
**BPE**	**0.08**
Abscellar methastasis present	0.64
Edema	0.11

Necrosis present	0.23
**Histopathological results**	
**Tumor type**	**0.04**
ER	0.06
**PGR**	**0.03**
**Ki-67**	**<0.01**
HER-2 Level	0.68
**Grade**	**<0.01**
